# Supplementary material for: The Effect of Eight-Week Functional Core Training on Core Stability in Young Rhythmic Gymnasts: A Randomized Clinical Trial
Source: Int J Environ Res Public Health. 2022 Mar 16;19(6):3509. doi: 10.3390/ijerph19063509 (PMC8953477; doi:10.3390/ijerph19063509)
Supplement: Supplementary file 1 [file ijerph-19-03509-s001.zip › ijerph-1599044-supplementary.pdf]

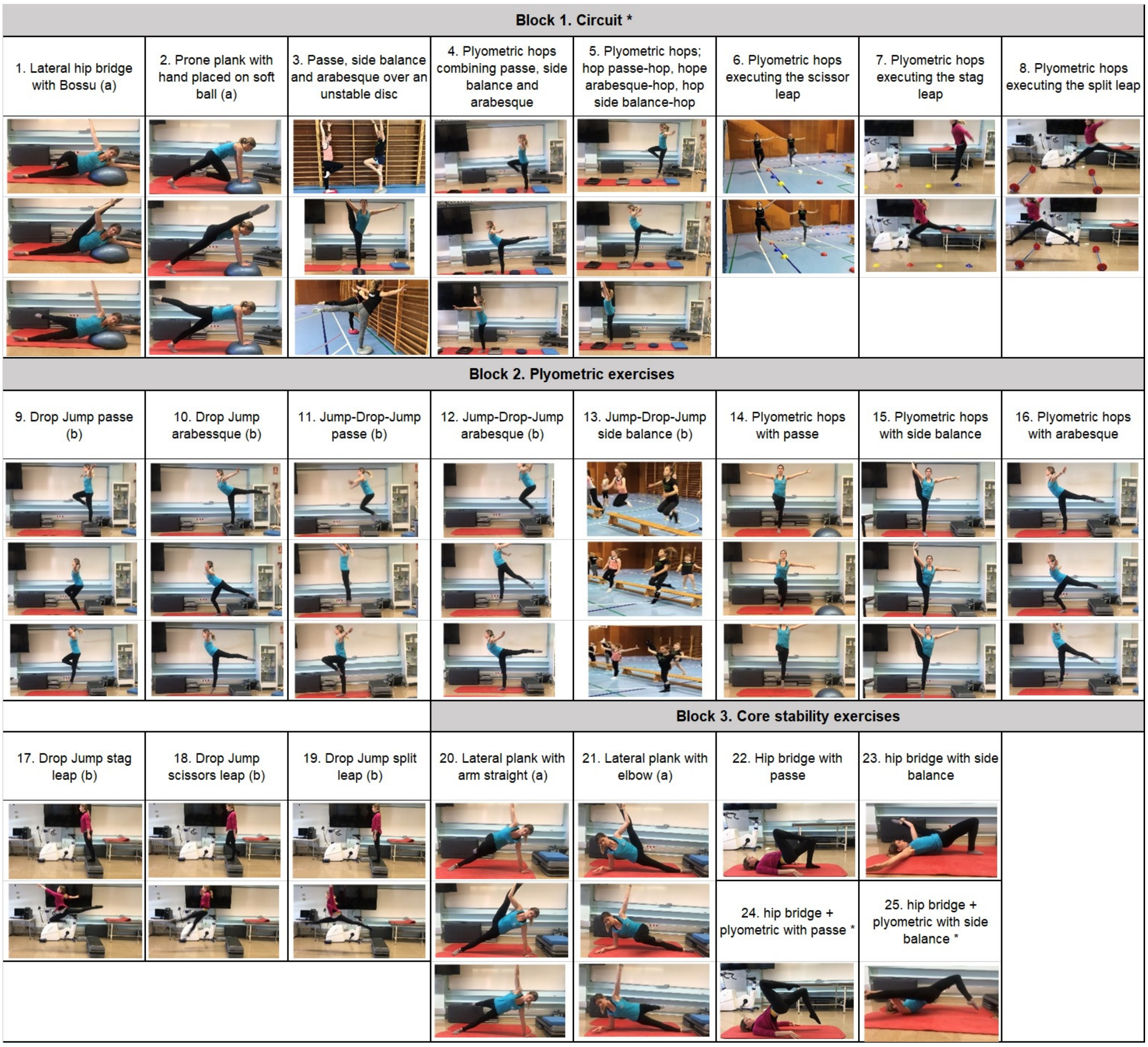


**Figure S1.** Training protocol exercises; all exercises were performed with right and left sides. * Mixed CS and plyometric exercises. (a) All planks and lateral hip bridges were executed with passe, side leg, and arabesque positions and were maintained for 2″ in each position. (b) All drop jumps and jump-drop jumps were performed with a 30 cm bench.

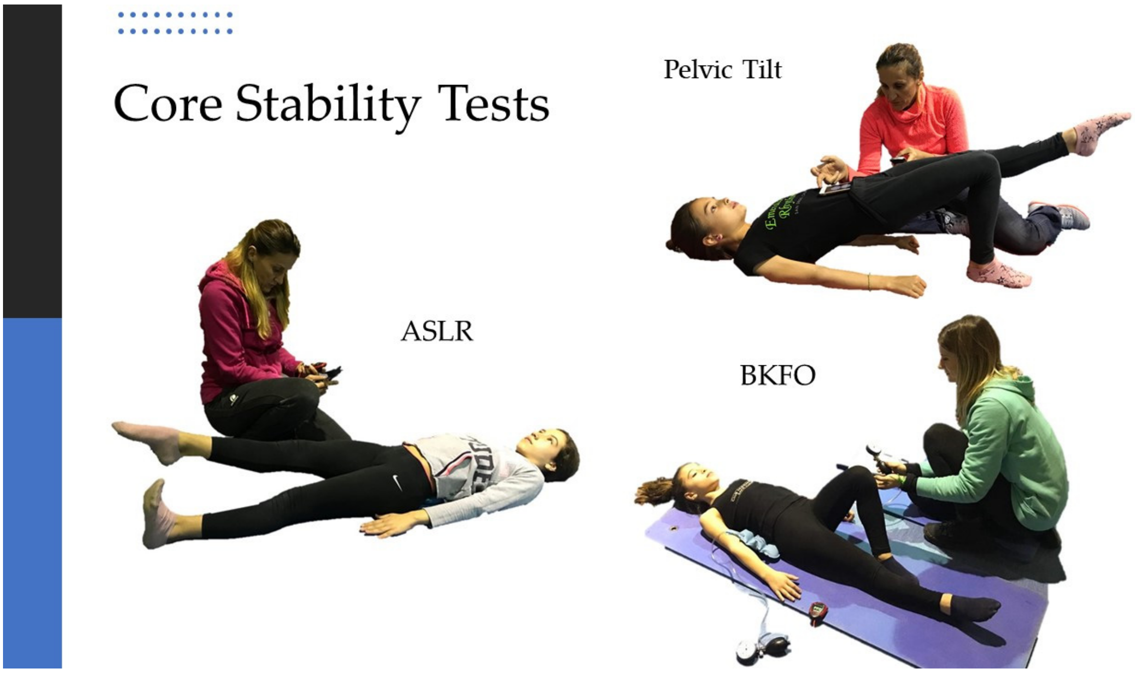


**Figure S2.** CS tests images; BKFO, ASLR and, Pelvic Tilt.

**Supplementary materials.** Weekly training content

| | **Tuesday** | | **Thursday** | | **Friday** | |
|---|---|---|---|---|---|---|
| hour | EG | CG | EG | CG | EG | CG |
| 6:00-6:30pm | Activation and stretching | Activation and stretching | Activation and stretching | Activation and stretching | Activation and stretching | Activation and stretching |
| 6:30-7:00pm | **CST Intervention** | RG warm-up | **CST Intervention** | RG warm-up | **CST Intervention** | RG warm-up |
| 7:00-7:15pm | Flexibility on benches | Flexibility on benches | Flexibility on benches | Flexibility on benches | Flexibility on benches | Flexibility on benches |
| 7:15-8:15pm | Ballet + Balances &turns | Ballet + Balances &turns | Acrobatics and leaps | Acrobatics and leaps | Apparatus handling | Apparatus handling |
| 8:15-9:00pm | Competition Choreographies | Competition Choreographies | Competition Choreographies | Competition Choreographies | Competition Choreographies | Competition Choreographies |

**Figure S3.** Weekly training content.
